# Nomogram construction and evaluation for predicting non-remission after a single radioactive iodine therapy for Graves’ hyperthyroidism: a retrospective cohort study

**DOI:** 10.3389/fendo.2024.1391014

**Published:** 2024-08-21

**Authors:** Feng Yu, Wenhui Ma, Xue Li, Ruiguo Zhang, Fei Kang, Weidong Yang, Renfei Wang, Jing Wang

**Affiliations:** ^1^ Department of Nuclear Medicine, Xijing Hospital, Air Force Military Medical University, Xi’an, China; ^2^ Department of Nuclear Medicine, Tianjin Medical University General Hospital, Tianjin, China; ^3^ Department of Nuclear Medicine, Shanghai Tenth People’s Hospital, Tongji University School of Medicine, Shanghai, China

**Keywords:** Graves’ hyperthyroidism, radioactive iodine therapy, non-remission, prediction model, nomogram

## Abstract

**Background:**

Radioactive iodine (RAI) therapy is a widely used treatment for Graves’ Hyperthyroidism (GH). However, various factors can impact the non-remission rate of GH after single RAI therapy. This study aimed to develop an online dynamic nomogram to assist physicians in providing personalized therapy for GH.

**Methods:**

Data from 454 GH patients who received RAI therapy were retrospectively reviewed and included in the present study. The univariate and multivariate analysis were conducted to investigate and identify independent influencing factors. The nomogram was developed based on the training cohort to explore non-remission rates. Finally, the reliability and accuracy of the constructed nomogram model were verified in the validation cohort via the calibration, receiver operating characteristic (ROC) curve, and decision curve analysis (DCA).

**Results:**

24-hours radioactive iodine uptake (RAIU_24h_), effective half-life (Teff), total iodine dose (TID) and iodine dose per gram of thyroid tissue (IDPG) were independent predictors. The nomogram had a high C-index 0.922 (95% CI: 0.892–0.953), for predicting non-remission. The calibration curves demonstrated excellent consistency between the predicted and the actual probability of non-remission. ROC analysis showed that the AUC of the nomogram model and the four independent factors in the training cohort were 0.922, 0.673, 0.760, 0.761, and 0.786, respectively. The optimal cutoff value for the total nomogram scores was determined to be 155. A total score of ≥155 indicates a higher likelihood of non-remission after a single RAI therapy for GH, whereas a score below 155 suggests a greater likelihood of remission. Additionally, the DCA curve indicated that this nomogram had good clinical utility in predicting non-remission.

**Conclusion:**

An online nomogram was constructed with good predictive performance, which can be used as a practical approach to predict and assist physicians in making personalized therapy decisions for GH patients.

## 1 Introduction

Graves’ Hyperthyroidism (GH), caused by Graves’ disease (GD), is a common endocrine disorder characterized by excessive thyroid hormone production (1–3). The treatment options for GH include antithyroid drugs, surgery, and Radioactive iodine (RAI) therapy. Antithyroid drugs (ATD), particularly methimazole, are currently considered the preferred treatment option (4–6). However, in certain circumstances such as liver disease, congestive heart failure, advanced age with comorbidities, or periodic paralysis, RAI may be preferred (4, 5).

For both patients and clinicians, the non-remission rate is a crucial factor in determining the choice of treatment. The relapse rates are 52-53% following ATD, 8-15% following RAI, and 0-10% following thyroidectomy (7, 8). Although, RAI therapy is a widely used treatment due to its effectiveness, convenience, and low price, various factors can impact the non-remission rate of GH. Therefore, it is important to identify the risk factors for non-remission and to develop strategies to minimize the probability of non-remission. However, to our knowledge, there is lack of study to develop a dynamic prediction model for non-remission in GH treated with RAI.

Nomogram is a valuable statistical tool that can transform predictive models into visual graphs, providing a numerical probability of a specific clinical event (9). Therefore, this study aimed to develop an online dynamic nomogram to assist physicians in providing a personalized therapy for GH.

## 2 Materials and methods

### 2.1 Patients

This retrospective analysis includes 129 patients treated at Xijing Hospital (from September 2017 to April 2022) and 325 patients treated at Tianjin Medical University General Hospital (from June 2017 to February 2019), all of whom were referred for RAI therapy for GH. GH was diagnosed based on a combination of clinical and laboratory criteria, including thyrotoxicosis, moderately diffuse goiter, elevated thyroid hormones, decreased thyrotropin (TSH), positive thyrotropin receptor antibody (TRAb), elevated radioactive iodine uptake (RAIU), or accompanied with exophthalmos or pretibial myxedema (4, 10). Thyroid glands >80 grams in size were not represented. The study cohort consisted of 454 GH patients, with a female-to-male ratio of 3.3:1. The average age was 41.8 ± 11.8 years.

#### 2.1.1 Patients’ preparation and RAI therapy

All patients were explained the procedure and precautions, including maintaining a low-iodine diet and avoiding iodide-containing medications for 7-14 days prior to treatment. Pregnant and breastfeeding patients were excluded. ATD was to be discontinued at least 3 days prior to RAI therapy.

Laboratory investigations including TSH, triiodothyronine (T3), thyroxine (T4), Free triiodothyronine (FT3), free thyroxin (FT4), Thyroglobulin antibody (TgAb), thyroid peroxidase (TPOAb) and TRAb levels with normal reference ranges as follow: TSH: 0.27-4.2 uIU/mL, T3:1.2-3.1 nmol/L, T4: 66-181 nmol/L, FT3: 3.1-6.8 pmol/L, FT4: 12-22 pmol/L, TgAb: 0-40 IU/mL, TPOAb: 0-35 IU/mL, TRAb: 0-1.5 IU/mL. Thyroid ultrasonography was done to detect the presence of nodules and measure the depth (D, cm), width (W, cm), and length (L, cm) of each thyroid lobe. The total thyroid volume (TV) was determined by summing the calculated products for both lobes, where the volume of one lobe was calculated as D×W×L. The thyroid mass (TM) was calculated with the formula (11): TM (g)=0.479×TV (cm^3^). Thyroid scan with 99mTechnetium-pertechnetate (99mTc) was done for all patients to exclude the presence of subacute thyroiditis. The RAIU of the thyroid was measured dynamically at 6, 24, 48, 72, and 144 hours to determine the values of 24-hours radioactive iodine uptake (RAIU_24h_), maximal radioactive iodine uptake (RAIUmax), and effective half-life (Teff).

After consultation with more than two nuclear medicine specialists, personalized ^131^I doses were calculated by following formula (11): $A=\frac{0.67\times \text{TM}\times \text{pAD}}{\text{Teff}\times \text{RAIUmax}}$ . Where: A— therapeutic radioactivity (mCi); 0.67—correction coefficient; TM—estimated thyroid mass (g); pAD—prediction absorption dose=100Gy/g.

#### 2.1.2 Assessment of therapeutic efficacy

All 454 patients were followed for 6 months to 1 year after receiving RAI therapy. The therapeutic efficacy was evaluated based on the following criteria (10). Euthyroidism was determined by the absence of symptoms or signs of GH and normal levels of FT3, FT4, and TSH in the serum. The diagnosis was hypothyroidism if a patient exhibited symptoms or signs of hypothyroidism (or without), with FT3 and FT4 levels below normal and TSH levels above average. Partial remission was characterized by alleviation of GH symptoms, partial resolution of signs, and reduction in serum levels of FT3 and FT4, although not reaching normal levels. No response was defined as either no significant improvement or an increase in symptoms and signs of hyperthyroidism, with no decrease in serum levels of FT3 and FT4. We categorized euthyroidism and hypothyroidism as “remission” (remission group) and persistent hyperthyroidism (including partial remission and no response) as “non-remission” (non-remission group).

### 2.2 Collection of variables

The patients’ clinical records, including age, gender, disease course, RAIU_24h_, RAIUmax, Teff, Thyroid weight, FT3, FT4, FT3/FT4, TSH, TRAb, TgAb, and TPOAb, total iodine dose (TID), iodine dose per gram of thyroid tissue (IDPG), were thoroughly reviewed. It should be noted that we transformed the continuous variables TRAb, TgAb, and TPOAb into more easily observable categorical variables based on the upper limits of their normal reference ranges and the upper measurable limits.

#### 2.2.1 Study design

To ensure the accuracy and clinical application efficiency of the established model, all patients were randomly divided into training and validation cohorts at a 7:3 ratio using R software. The patients were categorized based on their therapeutic efficacy and divided into two groups. Consequently, the training cohort was separated into group 1 (remission, n=217) and group 2 (non-remission, n=101). The validation cohort was separated into group A (remission, n=87) and group B (non-remission, n=49). Subsequently, univariate and multivariate analyses were conducted to investigate the effects of the variables. Moreover, a nomogram was developed based on the training cohort to predict the non-remission rates for GH patients, serving as a valuable tool in clinical practice. Finally, the predictive capability of the constructed nomogram was validated in the validation cohort.

### 2.3 Statistical analysis

The T-test was used to compare differences in age, Teff, RAIU_24h_, RAIUmax, TSH, TID, and IDPG, while the Mann-Whitney U test was used to compare differences in thyroid weight, FT3, FT4, and FT3/FT4. The chi-squared test was used to analyze differences in categorical variables. Multivariate analysis was used to identify independent factors associated with non-remission of Graves’ hyperthyroidism after RAI therapy. The performance of prediction and discrimination was evaluated using Harrell’s concordance index (C-index) (12), and the predictive power of the nomogram was shown using the ROC curve, with the area under the curve (AUC) values listed. The calibration curve was generated using the rms package in R language, which reflected the relationship between the predicted and actual incidence. The DCA curve was used to evaluate the clinical application value of the nomogram model by showing the net benefit of the observers. The nomogram was established using R software (version 4.0.5; http://www.rproject.org/), and all statistical analyses were performed using SPSS software version 25.0 (SPSS Inc Chicago, IL, USA).

## 3 Results

### 3.1 The baseline of clinical characteristics

**Figure 1:**
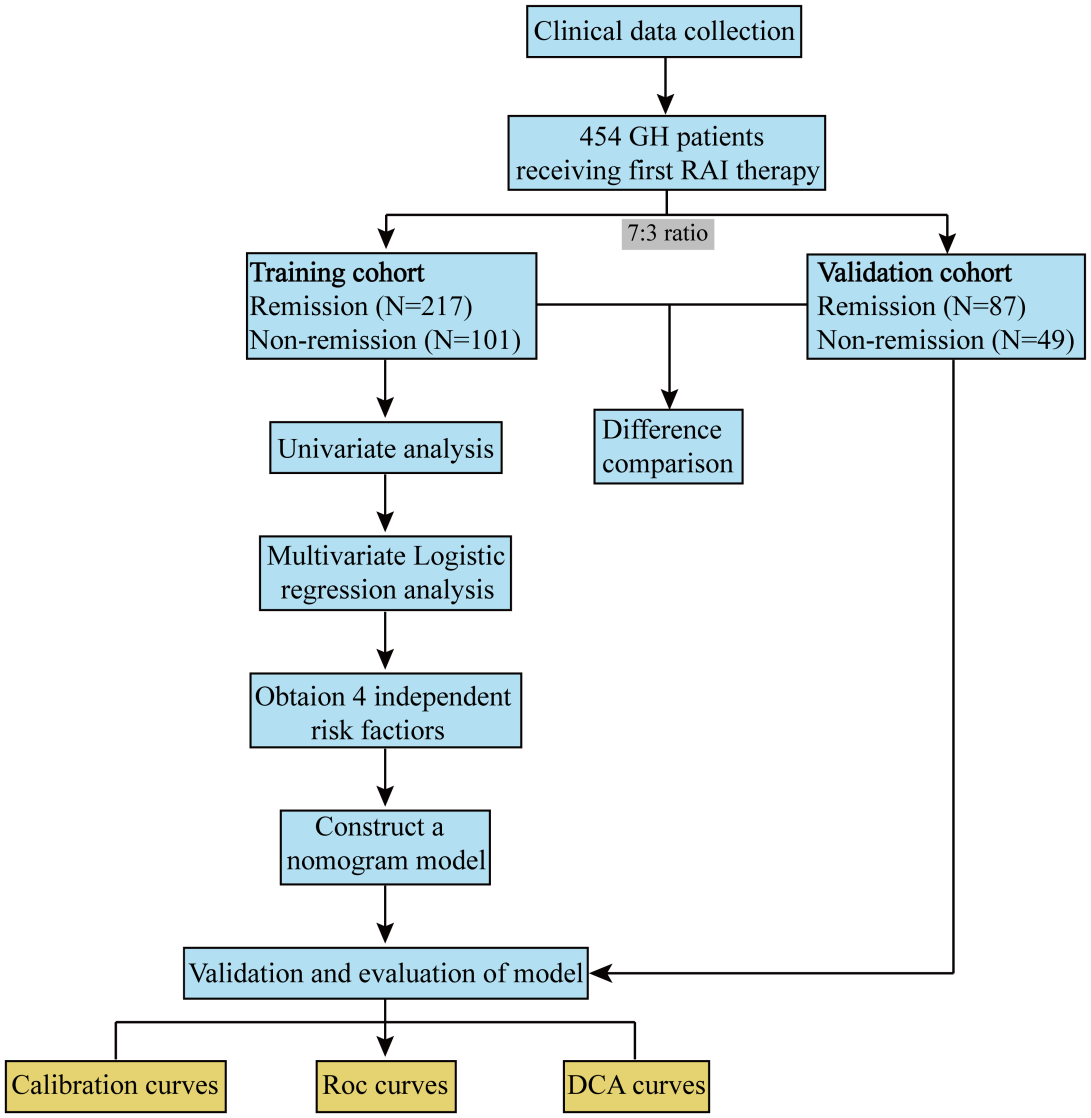
Flow chart.

During the study period, 454 patients with GH who met the inclusion criteria were included, with 318 and 136 patients assigned to the training and validation cohorts, respectively (**Figure 1**).

**Table 1** lists the clinical characteristics of the patients, which were similar between the training and validation cohorts. Non-remission after RAI therapy was observed in 103 (32.4%) and 47 (34.6%) patients in the two cohorts, respectively.

**Table 1:** Participant characteristics.

Variable	Cohort, No. (%)		t/Z/X^2^	P value
	Training (n=318)	Validation (n=136)		
Age	42.9 ± 14.1	43.6 ± 13.8	-0.511	0.609^a^
Gender				
male	71(22.3)	35(25.7)	0.618	0.432^b^
female	247(77.7)	101(74.3)		
Disease course (years)				
≤2	128(40.3)	57(41.9)	0.109	0.742^b^
>2	190(59.7)	79(58.1)		
Teff (d)	5.8 ± 1.5	5.7 ± 1.6	0.724	0.470^a^
RAIU_24h_ (%)	61.3 ± 11.0	61.9 ± 10.2	-0.555	0.579^a^
RAIUmax (%)	72.4 ± 10.5	72.6 ± 9.7	-0.228	0.820^a^
Thyroid weight (g)	40.3(20.0-78.9)	42.6(18.9-83.3)	-1.745	0.081^d^
FT3 (pmol/L)	18.6(11.0-33.1)	18.9(11.7-40.4)	-0.442	0.659^d^
FT4 (pmol/L)	37.5(27.2-51.2)	35.6(28.0-48.8)	-0.329	0.742^d^
FT3/FT4	0.492(0.386-0.691)	0.492(0.386-0.751)	-0.821	0.412^d^
TSH (uIU/mL)	0.031 ± 0.267	0.011 ± 0.055	0.849	0.396^a^
TRAb (IU/L)				
<1.5	9(2.8)	6(4.4)	1.086	0.786^c^
1.5-15	156(49.1)	64(47.1)		
15-40	100(31.4)	45(33.1)		
>40	53(16.7)	17(15.4)		
TgAb (IU/L)				
<40	174(54.7)	59(43.4)	5.181	0.075^b^
40-3000	100(31.4)	56(41.2)		
>3000	44(13.8)	21(15.4)		
TPOAb (IU/L)				
<35	91(28.6)	49(36.0)	2.472	0.290^b^
35-1000	135(42.5)	51(37.5)		
>1000	92(28.9)	36(26.5)		
TID (mCi)	9.1 ± 5.0	8.4 ± 3.8	1.464	0.144^a^
IDPG (µCi/g)	302 ± 140	320 ± 155	-1.191	0.234^a^
Efficacy				
remission	215(67.6)	89(65.4)	0.203	0.653^b^
non-remission	103(32.4)	47(34.6)		

Data are expressed as mean ± standard deviation, the median (percentiles 25-75) or frequencies.

^a^means T-test, ^b^means Pearson Chi-squared test, ^c^means Chi-squared test (fisher’s exact), ^d^means Mann-Whitney U test.

### 3.2 Risk factors for non-remission of GH after single RAI therapy in the training cohort

Upon analyzing the relationship between clinical characteristics and non-remission of GH after single RAI therapy, a total of 16 factors were involved in the univariate analyses (**Table 2**). The results showed that patients with shorter Teff, lower RAIU_24h_, higher FT3/FT4, higher TRAb level, lower TID and IDPG would have a higher probability of non-remission of GH (P<0.001, P<0.001, P=0.044, P<0.001, P<0.001, P<0.001, respectively). However, we found no statistically significant differences in age (P =0.398), gender (P =0.566), disease course (P =0.721), RAIUmax (P =0.146), thyroid weight (P =0.391), FT3 (P =0.631), FT4 (P =0.899), TSH (P =0.138), TgAb (P =0.110), and TPOAb (P =0.331).

**Table 2:** Univariate analysis in the training cohort.

Variable	Remission n=215	Non-remission n=103	t/Z/X^2^	P value
Age	43.4 ± 14.3	41.9 ± 13.7	0.846	0.398^a^
Gender				
male	50(23.3)	21(20.4)	0.330	0.566^a^
female	165(76.7)	82(79.6)		
Disease course (years)				
≤2	88(40.9)	40(38.8)	0.127	0.721^b^
>2	127(59.1)	63(61.2)		
Teff (d)	6.2 ± 1.3	4.8 ± 1.5	8.812	<0.001^a^
RAIU_24h_ (%)	63.4 ± 10.7	56.0 ± 10.4	5.085	<0.001^a^
RAIUmax (%)	71.2 ± 11.0	72.8 ± 8.0	-1.456	0.146^a^
Thyroid weight (g)	40.1(21.1-72.4)	42.2(18.3-75.8)	-0.858	0.391^d^
FT3 (pmol/L)	18.6(11.0-30.8)	18.8(11.5-46.1)	-0.480	0.631^d^
FT4 (pmol/L)	37.4(27.0-51.8)	37.6(28.3-48.6)	-0.126	0.899^d^
FT3/FT4	0.466(0.370-0.649)	0.534(0.409-0.716)	-2.012	0.044^d^
TSH (uIU/mL)	0.009 ± 0.048	0.077 ± 0.463	-1.497	0.138^a^
TRAb (IU/L)				
<1.5	6(2.8)	3(2.9)	17.009	0.001^c^
1.5-15	119(55.3)	37(35.9)		
15-40	66(30.7)	34(33.0)		
>40	24(11.2)	29(28.2)		
TgAb (IU/L)				
<40	116(54.0)	48(51.6)	4.423	0.110^b^
40-3000	74(34.4)	26(28.0)		
>40	25(11.6)	19(20.4)		
TPOAb (IU/L)				
<35	57(26.5)	34(33.0)	2.208	0.331^b^
35-1000	97(45.1)	38(36.9)		
>1000	61(62.2)	31(30.1)		
TID (mCi)	10.1 ± 4.7	7.1 ± 4.9	5.227	<0.001^a^
IDPG (µCi/g)	340 ± 142	222 ± 94	8.857	<0.001^a^

Data are expressed as mean ± standard deviation, the median (percentiles 25-75) or frequencies.

^a^means T-test, ^b^means Pearson Chi-squared test, ^c^means Chi-squared test (fisher’s exact), ^d^means Mann-Whitney U test.

Multivariate logistic regression analysis using a stepwise method was performed based on related factors from the univariate analysis. The results revealed that a total of 4 factors, including RAIU_24h_, Teff, TID, IDPG, were verified as independent risk factors that predicted non-remission of GH after RAI therapy. On multivariate analysis, with results reported as odds ratio (95% CI), subjects with RAIU_24h_ (0.910 [0.874-0.947]), Teff (0.362 [0.262-0.501]), TID (0.777 [0.702-0.959]) and IDPG (0.982 [0.976-0.987]) had a higher probability of non-remission of GH after RAI therapy (**Table 3**).

**Table 3:** Multivariate analyses in the training cohort.

Variable	β	OR	95%CI	P value
RAIU_24h_ (%) (X_1_)	-0.094	0.910	0.874-0.947	<0.001
Teff (d) (X_2_)	-1.015	0.362	0.262-0.501	<0.001
TID (mCi) (X_3_)	-0.253	0.777	0.702-0.959	<0.001
IDPG (µCi/g) (X_4_)	-0.018	0.982	0.976-0.987	<0.001
Constant	17.530	0.000		

### 3.3 Construction of a prediction model of non-remission of GH after a single RAI therapy

**Figure 2:**
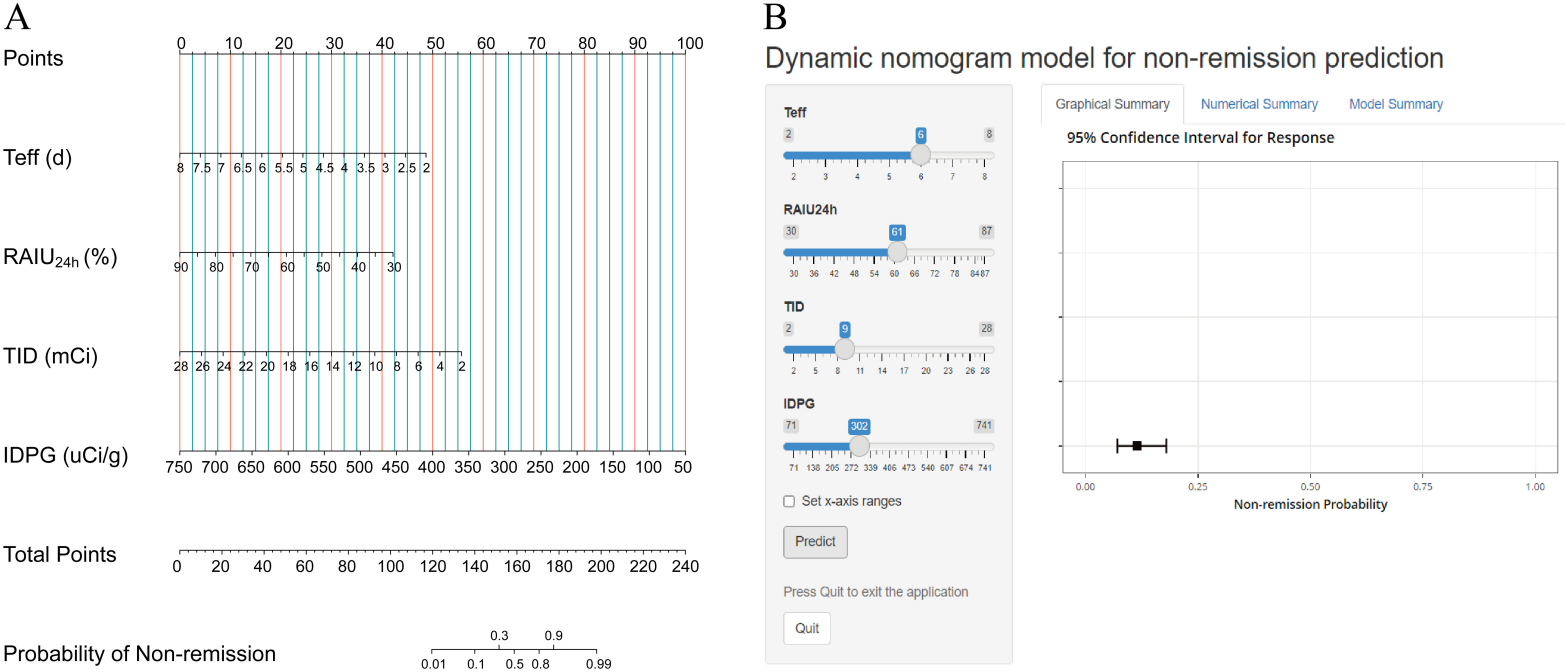
Nomogram prediction model for non-remission probability. **(A)** Established nomogram in the training cohort incorporating the following four parameters: Teff, RAIU24h, TID and IDPG. **(B)** Online dynamic nomogram accessible at https://yufengyxlxt.shinyapps.io/DynNom_model/.

Based on the multivariate analysis results, the independently associated risk factors were used to form a simple-to-use nomogram model for predicting non-remission probability, which is illustrated in **Figure 2A** and available online (https://yufengyxlxt.shinyapps.io/DynNom_model/) and presented in **Figure 2B**. Each variable was assigned a score on a scale. A total score was obtained by adding scores for each selected variable. A vertical line was then drawn along the total points series to estimate the risk of non-remission of GH after RAI therapy. The prediction model showed that IDPG was the top 1 contributor to non-remission, followed by TID, Teff and RAIU_24h_ (**Figure 2A**).

### 3.4 Evaluation of the nomogram model

**Figure 3:**
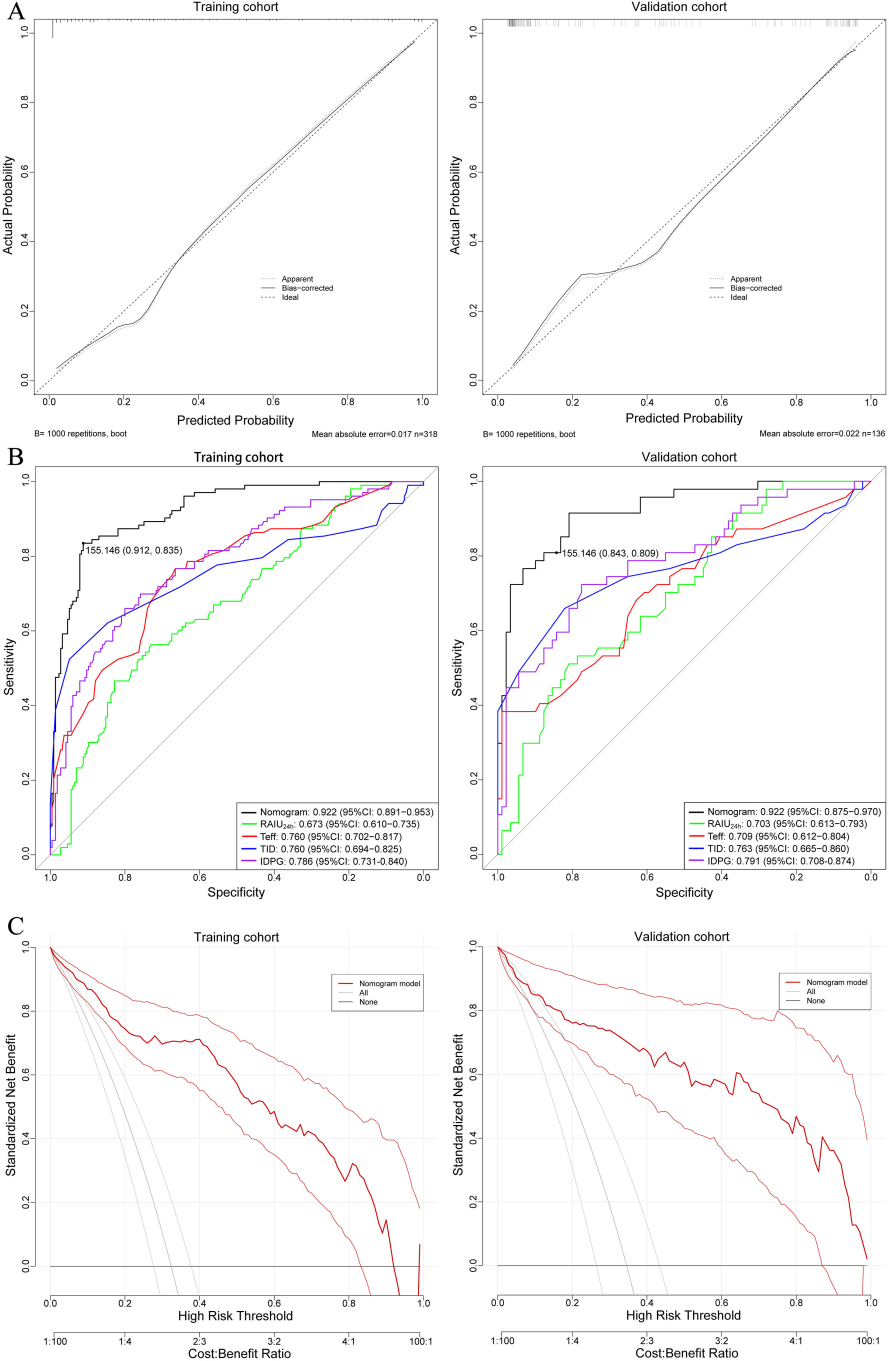
**(A)** Calibration curves showing the agreement between predicted and observed probabilities of non-remission, for both training and validation cohorts. The closer the curve aligns with the diagonal dashed line (ideal calibration line), the better the predictive accuracy. **(B)** ROC curves of the nomogram prediction for non-remission probability of the training and validation cohorts. **(C)** Decision curve analysis (DCA) illustrating the clinical usefulness of the nomogram for predicting non-remission probability in the training and validation cohorts. DCA assesses the net benefit of using the nomogram across a range of threshold probabilities, aiding clinical decision-making.

The nomogram exhibited a high C-index of 0.922 (95% CI: 0.892–0.953) for predicting non-remission of GH following RAI therapy, indicating a high level of accuracy in the model’s predictions. Calibration curves demonstrated excellent consistency between the predicted results and the actual probability of non-remission in GH patients after RAI therapy (**Figure 3A**). ROC analysis revealed that the AUC of the nomogram model and the four independent factors (including RAIU24h, Teff, TID, and IDPG) in the training cohort were 0.922 (95% CI: 0.891–0.953), 0.673 (95% CI:0.610-0.735), 0.760 (95% CI: 0.702-0.817), 0.760 (95% CI: 0.694-0.825), and 0.786 (95% CI: 0.731-0.840), respectively. The five models could be ranked from best to worst as follows: nomogram model > IDPG > TID > Teff > RAIU24h (**Figure 3B**). Similar results were observed in the validation cohort. Additionally, the DCA curve indicated that this nomogram had good clinical utility in predicting non-remission of GH after RAI therapy (**Figure 3C**). The optimal cutoff value for the total nomogram scores was determined to be 155, with specificity and sensitivity of 91.2% and 83.5% in the training cohort, and 84.3% and 80.9% in the validation cohort, respectively (**Figure 3B**).

## 4 Discussion

Over the years, RAI therapy has been one of the most important methods for treating GH (4). Many physicians prefer to use a large dose of the isotope to achieve early hypothyroidism and avoid the need for another dose of iodine-131. However, there are still 8-15% of patients who do not achieve complete remission after the initial treatment and require a second round of RAI therapy 3-6 months later. Numerous studies have been conducted to determine the ideal dosage of iodine-131 that would increase the likelihood of achieving euthyroid in patients. However, these studies often overlooked other predictive factors that could influence the treatment outcome (13–15). In our study, we proposed four predictive factors: RAIU_24h_, Teff, TID, and IDPG, and used these four factors to construct a nomogram model for predicting non-remission after the first RAI therapy.

RAIU_24h_ plays a crucial role in determining the iodine-131 dose and is included in various iodine-131 dose calculation formulas. Numerous studies have been conducted to investigate the relationship between RAIU_24h_ and the efficacy of RAI therapy, both domestically and internationally. In our study, we observed patients with high RAIU_24h_ had better outcomes with RAI therapy, which aligns with the conclusions of studies such as NordykeRA and NordykeRA, where higher RAIU_24h_ was associated with higher cure rates (16–18). Contrary to our findings, some studies have shown a negative correlation between the cure rate of RAI therapy for GH and RAIU_24h_, indicating that higher RAIU_24h_ is associated with lower cure rates (15, 19–21). There are several possible explanations for this phenomenon. Firstly, there may be an inverse relationship between the sensitivity of thyroid tissue to radiation and RAIU_24h_, as demonstrated in the study by Di Martino F et al (22). Secondly, the iodine-131 dose for treatment decreases as RAIU_24h_ increases, and low therapeutic dose of iodine-131 may reduce the uptake of iodine-131 in thyroid tissue (23, 24). Despite the inconsistency in results, RAIU_24h_ is still considered an important factor influencing the success of RAI therapy.

Teff refers to the time required for the radioactive nuclide iodine-131 to decrease to half of its original amount in the body, due to the combined effects of physical decay and biological activity. It reflects the duration of iodine-131 residence in thyroid tissue, which directly affects the actual absorbed dose of beta radiation in thyroid tissue, thereby influencing the extent of damage to the thyroid tissue by iodine-131. Yu et al. (11) found that a longer Teff increases the likelihood of developing hypothyroidism, while other study (25) reported no significant difference in Teff between the remission and non-remission groups after RAI therapy. In this study, both univariate and multivariate logistic regression analyses found that shorter Teff is associated with non-remission, which is consistent with the current general view. Shorter Teff reflects a high metabolic state of thyroid cells, resulting in a shorter residence time and lower absorbed dose of thyroid tissue (26).

The IDPG and TID can impact the actual absorbed dose of the thyroid tissue and therapeutic effect of RAI. Previous studies have shown that lower do IDPG result in higher failure rates (27). In both the univariate and multivariate logistic regression analyses in this study, the TID and IDPG identified as positive risk factors affecting treatment outcomes. This is consistent with the general consensus (28). We speculate that when higher TID, especially higher IDPG, are administered, it ensures sufficient beta radiation within the thyroid tissue to exert a destruction, resulting in satisfactory treatment outcomes. Currently, the typical IDPG in clinical practice is 70-150 µCi/g, but evidence suggests that in order to achieve a hypothyroid state in patients with GH, the IDPG needs to be greater than 150 µCi/g (29, 30). The ATA guideline recommends an IDPG of 50-200µCi in clinical practice, while the Chinese guideline suggests 70-150µCi for IDPG (4, 10). However, there is evidence suggesting that in order to achieve hypothyroid state, the IDPG needs to be greater than 15 MBq (405 µCi) or dosimetry aiming at delivering at least 300 Gy to the thyroid tissue for functional ablation (31, 32). Peters’ research has shown that RAI efficacy is demonstrated for glands up to 80 ml, and it is important to note that the patients presented in our study fit within previously shown gland sizes for RAI efficacy (<80 gm). In this study, glands were in the modestly enlarged range, and glands >80 gm were generally excluded. These findings are in agreement with those reported by Peters (31, 32). It is worth noting that in **Table 2**, the TID range in the validation group was 9.1 ± 5.0 mCi, which is notably lower than the commonly used fixed dose of 555 MBq (15 mCi). This preference for lower treatment doses is influenced by patient-centered or cultural considerations, aiming to restore euthyroidism rather than induce hypothyroidism in the initial management of GH using single RAI therapy. The choice of treatment target may differ from practices in other regions, such as the United States, where the standard approach is to use RAI doses that induce hypothyroidism. It should be emphasized that using lower doses aimed at restoring euthyroidism may result in lower long-term remission rates and could potentially limit the applicability of the model.

Through univariate and multivariate analyses, these four factors were ultimately identified as independent factors affecting the non-remission rate after initial RAI therapy in GH patients. However, in order to more accurately predict the probability of non-remission, we did not choose to use a single independent factor for prediction, but instead combined these four factors to construct a more predictive nomogram model. As shown in ROC curve analysis, it can be clearly seen that the nomogram model performed better in terms of predictive ability compared to single independent factor, both in the training set and validation set. Additionally, the nomogram model also demonstrated excellent consistency and clinical net benefit in calibration curve and DCA curve analysis. By applying this model, we can guide the adjustment of dosage before RAI administration to achieve a lower rate of non-remission and reduce the chances of a second RAI therapy.

However, it is important to acknowledge the limitations of our study. Firstly, this was a retrospective study with some inevitable bias. Secondly, the sample size of two center was small. The novel nomogram model was only validated internally. Therefore, a multicenter clinical study with a larger sample size could be conducted for external validation to verify the clinical benefits in the subsequent research. Thirdly, the patients included in this study had moderately enlarged thyroid glands, not markedly enlarged glands. This may limit the applicability of our model in treating GH patients with very large goiters. Nevertheless, this novel nomogram model does have the potential to improve clinical practice of personalized therapy.

## 5 Conclusion

This study was the first to develop and validate online nomogram based on the independent risk factors to dynamically predict non-remission probability in individuals with GH after initial RAI therapy. This novel model demonstrated superior performance and discriminative power, providing vital information for nuclear medicine physicians to design customized clinical treatments for their patients.

## Data Availability

The raw data supporting the conclusions of this article will be made available by the authors, without undue reservation.
